# The heterogeneity of cellular metabolism in the tumour microenvironment of hepatocellular carcinoma with portal vein tumour thrombus

**DOI:** 10.1111/cpr.13738

**Published:** 2024-08-27

**Authors:** Xiu‐Ping Zhang, Wen‐Bo Zou, Zhen‐Qi Li, Ze‐Tao Yu, Shao‐Bo Yu, Zhao‐Yi Lin, Fei‐Fan Wu, Peng‐Jiong Liu, Ming‐Gen Hu, Rong Liu, Yu‐Zhen Gao

**Affiliations:** ^1^ Faculty of Hepato‐Biliary‐Pancreatic Surgery, The First Medical Center of Chinese People's Liberation Army (PLA) General Hospital Medical School of Chinese PLA Beijing China; ^2^ Department of General Surgery No.924 Hospital of PLA Joint Logistic Support Force Guilin China; ^3^ Department of Clinical Laboratory Sir Run Run Shaw Hospital of Zhejiang University School of Medicine Zhejiang Hangzhou China; ^4^ The First Clinical Medical School Lanzhou University Lanzhou China; ^5^ Harbin Institute of Technology Harbin China

## Abstract

Given the growing interest in the metabolic heterogeneity of hepatocellular carcinoma (HCC) and portal vein tumour thrombus (PVTT). This study comprehensively analysed the metabolic heterogeneity of HCC, PVTT, and normal liver samples using multi‐omics combinations. A single‐cell RNA sequencing dataset encompassing six major cell types was obtained for integrated analysis. The optimal subtypes were identified using cluster stratification and validated using spatial transcriptomics and fluorescent multiplex immunohistochemistry. Then, a combined index based meta‐cluster was calculated to verify its prognostic significance using multi‐omics data from public cohorts. Our study first depicted the metabolic heterogeneity landscape of non‐malignant cells in HCC and PVTT at multiomics levels. The optimal subtypes interpret the metabolic characteristics of PVTT formation and development. The combined index provided effective predictions of prognosis and immunotherapy responses. Patients with a higher combined index had a relatively poor prognosis (*p* <0.001). We also found metabolism of polyamines was a key metabolic pathway involved in conversion of metabolic heterogeneity in HCC and PVTT, and identified ODC1 was significantly higher expressed in PVTT compared to normal tissue (*p* =0.03). Our findings revealed both consistency and heterogeneity in the metabolism of non‐malignant cells in HCC and PVTT. The risk stratification based on cancer‐associated fibroblasts and myeloid cells conduce to predict prognosis and guide treatment. This offers new directions for understanding disease development and immunotherapy responses.

## Graphical abstract

Graphical Abstarct

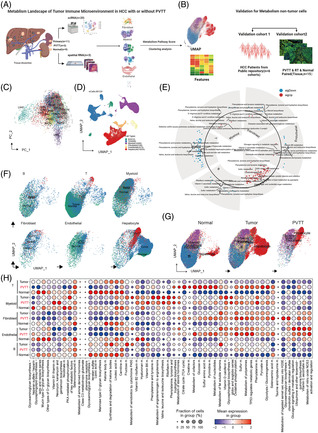

## 1 INTRODUCTION

Hepatocellular carcinoma (HCC) is increasingly threatening human life and health and imposes a serious disease burden worldwide.^1^ Despite advances in diagnostic and treatment strategies for HCC and its complications, it has a poor prognosis and low long‐term survival rate compared to other malignant.^2^ Portal vein tumour thrombus (PVTT) remains a common presentation in patients with HCC and is an important risk factor for poor prognosis. Approximately 30%–50% of patients diagnosed with HCC for the first time have concomitant PVTT.^3^ Continuously evolving treatment approaches, including surgical resection, regional interventional therapy, chemotherapy, radiotherapy, and combination therapy, have improved the long‐term survival of HCC patients with PVTT.^4^, ^5^ However, the poor therapeutic efficacy of treatments causes high mortality, and recurrence rates in HCC patients with PVTT remain high level.^6^, ^7^ Currently, the metabolism‐related signatures in HCC patients with PVTT are expected to become key therapeutic targets and have attracted much attention from researchers.^8^, ^9^

The development of malignant tumours, including HCC, requires plenty of nutrients to maintain rapid growth and escape immune attack.^10^ The convergence of metabolic adaptations creates fundamental competition for nutrients required by cancer cells and other non‐malignant cells within the tumour microenvironment (TME). Several studies have demonstrated that tumour initiation, progression, metastasis, and immune escape require metabolic reprogramming of cancer cells.^11^, ^12^ However, current progress in targeting cancer metabolism has been limited. Strategies for targeting the intrinsic metabolism of cancer cells often do not account for the metabolism of non‐malignant stromal and immune cells, which also play pivotal roles in tumour progression and drug resistance.^13^, ^14^ For example, cancer‐associated fibroblasts (CAFs) and adipocytes can support malignant cells by providing nutrients, such as alanine and lipids within the TME.^15^ Tumour‐associated macrophages (TAMs), which are the most important non‐malignant components of the TME, affect tumour progression via multiple metabolic pathways and are considered novel therapeutic target.^16^ Additionally, subtype transformation of CD8+ T cells can regulate the immune response by partaking in metabolic reprogramming.^17^ An increasing number of studies have revealed that metabolic reprogramming of non‐malignant cells can affect the immune status of malignant tumours.^18^, ^19^ Research has shown that TME and energy metabolic pathways, such as fatty acid biosynthesis, are involved in regulating the invasion and progression of PVTT.^8^ However, the corresponding compositional changes and regulatory mechanisms of the TME in PVTT still require further investigation. Therefore, studying metabolic changes in non‐malignant cells during the formation and development of PVTT may be a new breakthrough.

Herein, we systematically depicted the first comprehensive metabolic landscape of non‐malignant cells and explored their metabolic heterogeneity at single‐cell, spatial, and transcriptomic levels in the TME of HCC and PVTT. Notably, we identified and validated the key roles of metabolism of polyamines and ODC1 in the conversion of metabolic heterogeneity by combining spatial distribution analysis and multiplex immunohistochemistry (mIHC) technology. It will also provide new prospects for identifying targeted metabolic pathways or combined drugs for PVTT treatment.

## 2 MATERIALS AND METHODS

### 2.1 Study design and single‐cell RNA sequencing acquirements

**FIGURE 1:**
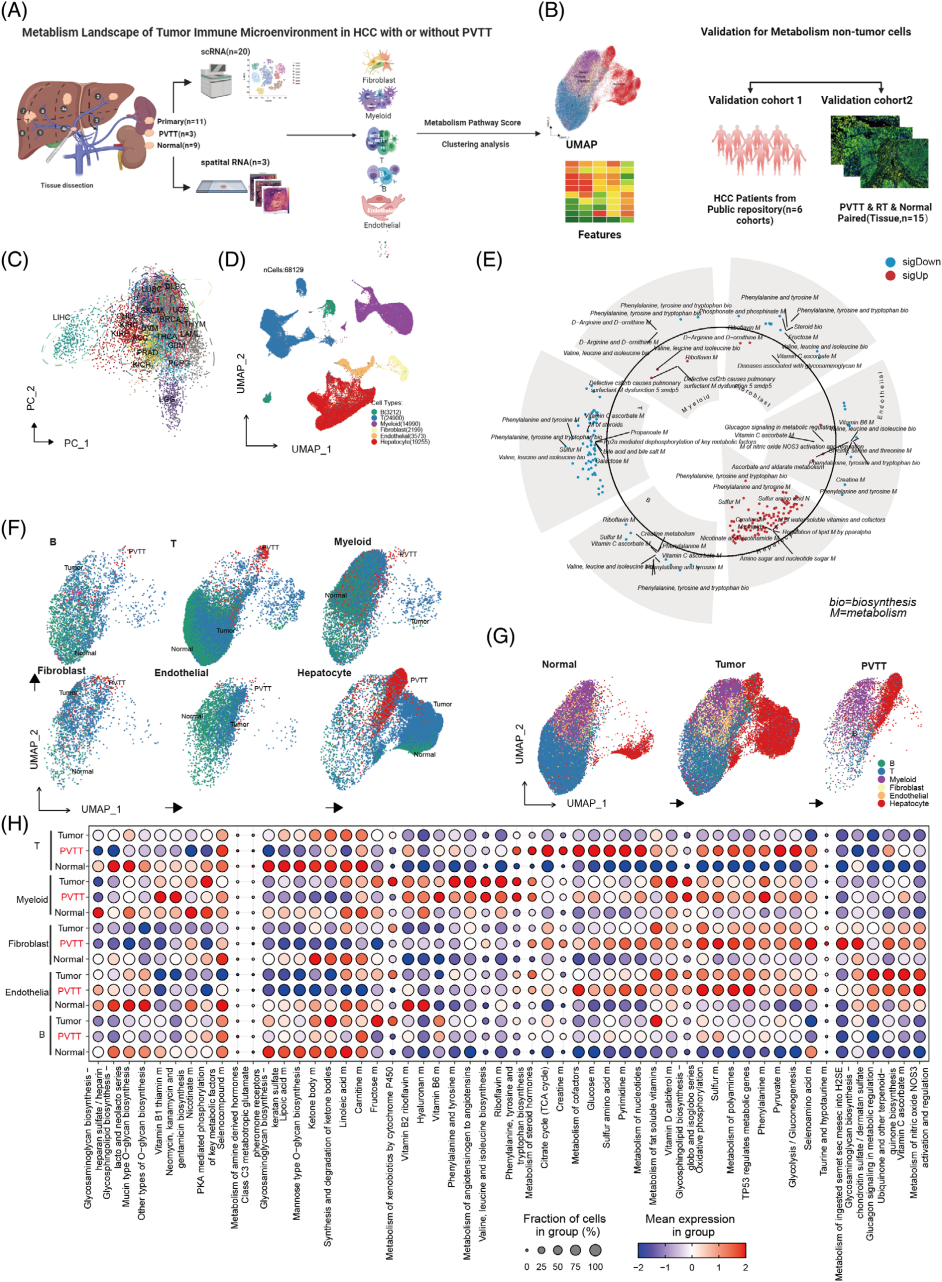
Metabolic Heterogeneity in the Tumour Microenvironment of HCC with PVTT. (A) and (B) Design flowcharts for studying metabolic heterogeneity in PVTT. (C) Enhanced metabolic characteristics in LIHC compared with other tumours. (D) Single‐cell cohort consisting of six primary cell types. (E) Variations in metabolic pathway enrichment across different cell types. (F) UMAP plots illustrating metabolic scores in B, T, Myeloid, Fibroblast, Endothelial, and Hepatocyte cells. (G) UMAP plots of metabolic scores in the normal, tumour, and PVTT samples. (H) Heterogeneity of metabolic scores in different cells from various sources, based on an analysis of tissue‐specific differences.

We obtained and comprehensively analysed 68,129 single‐cell RNA (scRNA) sequencing data encompassing six major cell types (T, B, myeloid, endothelial cells, fibroblasts, and hepatocytes) from the GSE149614 dataset in the Gene Expression Omnibus (GEO) database (https://ncbi.nlm.nih.gov/geo/). The corresponding search term was referred to as ‘Hepatocellular Carcinoma’, ‘portal vein tumor thrombus’, and ‘Single‐cell’. These cells were isolated from 20 patient samples, including primary HCC (*n* = 10), PVTT (*n* = 2), and adjacent normal liver tissues (*n* = 8). The flowchart of the present study is displayed in Figure 1A, B. Non‐malignant cells of the scRNA sequencing data were extracted from 20 samples. Subsequently, we clustered CAFs, myeloid, T, B, and endothelial cells into optimal subtypes using the cluster method. In addition, we retrieved five public RNA transcriptomics sequencing datasets and the corresponding prognostic information from the GEO database (GSE14520, GSE10143, GSE76427, and GSE15654) and the ICGC database (ICGC‐LIRI‐JP), as well as pan‐cancer cohorts, including bulk RNA sequencing data of 32 cancers from the Xena database (https://xenabrowser.net). To validate these results, we retrieved the spatial transcriptomics sequencing cohort (*n* = 3) from a public repository and prospectively collected five pairs of samples from the Chinese People's Liberation Army General Hospital for mIHC. The use of all human samples was approved by the Institutional Ethics Committee of the Chinese People's Liberation Army General Hospital. Informed consent was obtained from all patients.

### 2.2 Cell annotation of metabolic pathway score

Due to the sheer volume of dimensions, directly performing dimensionality reduction on high‐dimensional gene expression data is a challenge. Thus, we employed the scMetabolism package (version 2.1.0, https://github.com/wu-yc/scMetabolism) to calculate metabolic pathway scores for all collected scRNA sequencing data and further implemented REACTOME and KEGG analyses. We condensed the expression information for thousands of genes into hundreds of pathway activity scores. These scores provide a summary of the metabolic activities, making it easier to perform dimensionality reduction and subsequent biological interpretation. To ascertain the metabolic pathway scores (*n* = 162) for bulk RNA sequencing data, we compiled gene lists corresponding to identical numbers of metabolic pathways from the MSiDB database. These gene lists were analysed using the gene set variation analysis (GSVA) method. In the present study, the metabolic pathway scores of all HCC‐related samples and pan‐cancer cohorts were generated using this method.

### 2.3 Metabolism clustering for TME cells and tissue distribution preferences analysis

The Seurat package (version 5.1.0) was used to process the scRNA sequencing data based on the metabolism assay. Adhering to established protocols for scRNA sequencing data analysis, we implemented normalization, principal component analysis (PCA), and the FindNeighbors algorithm, followed by setting the resolution parameter of the FindClusters function at 0.3. This approach enabled effective clustering of the major cell types into 3–7 distinct meta‐clusters based on their metabolic pathway scores. Then, to delineate the tissue distribution of meta‐clusters, odds ratio (OR) values were calculated to analyse the tissue distribution of meta‐clusters. For each meta‐cluster *i* and tissue *j*, a 2 × 2 contingency table was used, and Fisher's exact test was used to determine OR and *p*‐values, which were also adjusted using the BH method. ORs >1.5 or <0.5 indicated significant tissue preferences.^20^

### 2.4 Differential expression analysis of metabolism pathway scores and genes

To elucidate the metabolic profile characteristics of each meta‐cluster, we isolated individual cell types for comprehensive analysis. We established a *p*‐value < 0.001 as the threshold to identify significantly differential expression of metabolic pathway scores among these cells. Next, we defined a minimum percentage (min.pct) of 0.25 and a log‐fold change (logfc) threshold of 0.25 for differentially expressed genes (DEGs) in the RNA assay. A network correlation was then constructed to examine the interrelationships between the top 10 genes and the top 3 most specific metabolic pathways in the distinct cell clusters using the network igraph package (version 2.0.3).

### 2.5 Signature of HCC‐related features for TME cells

To assess the features of these cells within various metabolic clusters, we used pre‐established gene lists for scoring. There are two primary methods for gene set acquisition. First, in the analysed scRNA sequencing cohort, we leveraged the original clustering method to identify differential genes, selecting the top 50 genes as representative signatures of cell features. Second, we gathered extensive data from public datasets. In this study, multiple public HCC datasets were compiled. Specifically, a set of 43 genes list associated with HCC prognosis was extracted from the MSiDB database (https://www.gsea-msigdb.org/gsea/index.jsp) using the keyword ‘HCC,’ which was detailed in the supplementary table. Additionally, we included an immune‐related gene set derived from a prior publication.^21^ The third CAFs gene set was obtained from pan‐cancer CAFs scRNA sequencing analysis and can be divided into four subtypes: pan.pCAF(proliferation), pan.dCAF(collagen), pan.myCAF(smooth muscle), and pan.iCAF(inflammation).^22^, ^23^ Finally, the fourth gene set related to macrophages was obtained from a previous publication.^24^

### 2.6 SCENIC and cellular communication analysis

In this study, we employed the pySCENIC Docker package (version 0.9.1), a Python‐based tool, to explore the gene regulatory networks (GRNs) of transcription factors (TFs) across various cell types categorized based on their metabolic pathway scores. We utilized two gene‐motif rankings—‘hg19‐tss‐centred‐10 kb’ and ‘hg19‐500 bp‐upstream’—from the RcisTarget database to identify transcription start sites and GRNs within the scRNA sequencing data. Next, we conducted a cellular communication analysis across different cell types using the CellPhoneDB package (version 4.1.0) in Python. CellPhoneDB is one of the most widely used software packages in Python to explain communication between cells and can distinguish between ligands and receptors and classify them into different categories, such as chemokines, costimulatory, and coinhibitory. The corresponding generation of TFs and ligand‐receptor pairs for these cells was visualized using the R software.

### 2.7 Functional enrichment analysis

We subsequently performed functional enrichment analysis on each metabolic cell type using a list of the top 150 expressed genes. It incorporates Gene Ontology (GO) and Kyoto Encyclopedia of Genes and Genomes (KEGG) for functional assessments. A *q*‐value <0.05 was established to delineate significant statistics. The functional enrichment analysis results for various cell types were visualized using the ggplot2 package (version 4.2.3).

### 2.8 Prognosis performance analysis

All patients possessing comprehensive prognostic data were analysed to ascertain the prognostic significance of each metabolic cell type within TME. For each cell type, the top 50 genes were employed to calculate the GSVA scores in bulk RNA sequencing data.

The cut‐off values for these variables were established using the ‘survminer’ package (version 4.2.3), which facilitates the differentiation of distinctive differences.

### 2.9 Immunotherapy response prediction

To detect the predicted value of PVTT‐related cell types for immunotherapy efficacy, we obtained a total of 40 HCC patients using RNA sequencing data and Response Evaluation Criteria in Solid Tumours (RECIST) response data, in which all the patients had been accepted by the immune checkpoint inhibitor therapy (6 patients were responders, 29 patients were non‐responders, and others were not estimated, GSE140901). Next, we collected information on patients with other tumours to further check the predictive value of these cell types, including urothelial cancer, melanoma, and bladder cancer.^25^, ^26^, ^27^, ^28^, ^29^

### 2.10 Spatial analysis for expression of genes and cells

Spatial RNA transcript data were obtained from a previous publication.^30^ In the present study, we specifically focused on HCC‐2T, HCC‐2P, and HCC‐2N as exploration samples. We employed two methods to map the distribution of cell types in the spatial tissue. First, the AUCell method was used to generate specific cell scores based on the top 50 genes, which is consistent with the approach used in bulk RNA sequencing analysis. Gene or meta‐cluster scores were visualized using the SPATA2 package. Second, Cell2location, a Bayesian model capable of deciphering fine‐grained cell types within spatial transcriptomic data and constructing comprehensive cellular maps of different tissues, was employed to illustrate the co‐expression of genes and cells. We set the Cell2location model, which was trained by the complete scRNA sequencing dataset including six main cell types previously utilized in our analysis, to visualize the spatial distribution of specific cell types and genes.

### 2.11 Fluorescent mIHC and tissue imaging

Fifteen samples from five paired PVTT and primary cancer and normal tissues from HCC patients, used as experimental and control samples, respectively, were processed as formalin‐fixed paraffin‐embedded sections. The tissues were sectioned at a thickness of 2 μm. The sections were then baked at 65°C for 1 h, followed by deparaffinization in xylene for 10 min, which was repeated three times. Rehydration was performed using absolute ethyl alcohol for 5 min (repeated twice), 95% ethyl alcohol for 5 min, and 75% ethyl alcohol for 2 min. Subsequently, the slides were rinsed three times with distilled water. Heat‐induced epitope retrieval was conducted in a microwave oven, and the slides were immersed in boiling EDTA buffer (Alpha X Bio, Beijing, China) for 15 min. Blocking was performed using an antibody diluent (Alpha X Bio, Beijing, China).

The mIHC staining process involved the use of the following primary antibodies: CD8 (ab237709, Abcam, Cambridge, UK), SMA (AF1032, Affinity Biosciences, China), and ODC1 (28728‐1‐AP, Proteintech, USA), each incubated for 1 h at 37°C. This was followed by a 10‐min incubation at 37°C with Alpha X Polymer HRP Ms + Rb (Alpha X Bio, Beijing, China). Visualization was facilitated using an Alpha X 7‐Colour IHC Kit (Alpha X Bio, Beijing, China). Primary antibodies were linked to specific fluorophores: CD8 with AlphaTSA 520, SMA with AlphaTSA 570, and ODC1 with AlphaTSA 620. After staining, heat‐induced epitope retrieval was repeated to remove all antibodies, including primary and secondary antibodies. Finally, the slides were counterstained with DAPI for 5 min and mounted with Antifade Mounting Medium (I0052; NobleRyder, Beijing, China). Imaging was performed using an Axioscan7 (ZEISS, Germany).

### 2.12 Statistical analysis

The primary statistical methods employed in this study are described in detail in the corresponding methodology section. Routine statistical analyses, such as Spearman's correlation analysis and non‐parametric tests, were conducted for the cell score or metabolism score in each subgroup. For PVTT occurrence in HCC samples, receiver operating characteristic (ROC) analysis was used to evaluate the diagnostic values of the combined cell signatures. Cox regression analysis was performed to investigate the prognosis of each cell type. Logistic regression analysis was used to construct the combined‐index based meta‐cluster. The main statistical software used in this study included R version 4.2.3 and Python 3.9. Unless stated otherwise, a p value of 0.05 was considered the threshold for statistical significance.

## 3 RESULTS

### 3.1 Metabolic heterogeneity in the microenvironment of HCC and PVTT

Initially, we calculated the KEGG metabolic pathways in the pan‐cancer cohorts of TCGA by GSEA method, the results showed that HCC displayed markedly distinct metabolic profiles compared to other cohorts in the pan‐cancer cohort (Figure 1C). Subsequently, to observe the metabolic status of TME cells in HCC with PVTT, we annotated all single cells, categorizing them into B cells, T cells, CAFs, endothelial cells, myeloid cells, and hepatocytes (Figure 1D), in the 20 samples from 10 HCC and two PVTT patients using 162 metabolic pathways by the scMetabolism tool. Next, we observed significant differences in metabolic pathway scores among the six primary cell types (Supplemental Table S1). Metabolic diversity among non‐malignant cells in HCC, particularly in CAFs and myeloid cells, relative to hepatocytes, is shown in Figure 1E. The Uniform Manifold Approximation and Projection (UMAP) plot further corroborated this metabolic heterogeneity (Figure 1F). Intriguingly, cells from PVTT possessed distinct metabolic profiles compared with those from normal and tumour tissues (Figure 1G and Supplemental Table S2). The markedly different metabolic pathway activations in non‐malignant cells across HCC, PVTT, and normal tissues are shown in Figure 1H and Supplemental Table S3. The metabolic UMAP for each patient is shown in Supplementary Figure S1A. Building on this observation, we categorized all non‐malignant cells into distinct metabolic meta‐clusters, as depicted in Supplementary Figure S1B. The bar plot for an individual sample of these meta‐clusters is illustrated in Supplementary Figure S1C. In summary, our initial analysis revealed significant metabolic heterogeneity among non‐malignant cells within the HCC microenvironment, particularly concerning PVTT, warranting further investigation.

### 3.2 PVTT tissue‐specific metabolic CAFs exhibit strong cell communications

**FIGURE 2:**
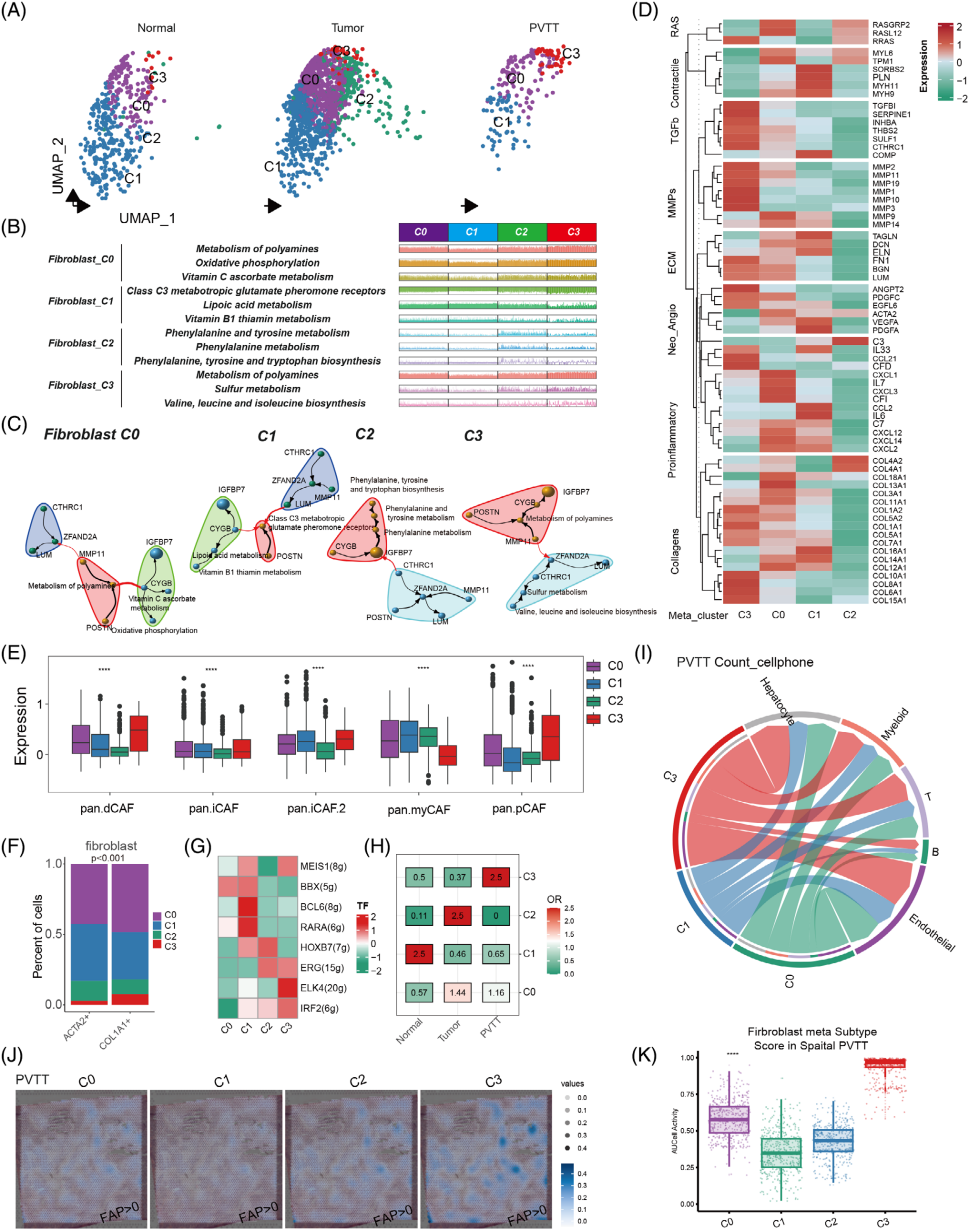
Tissue metabolic heterogeneity in fibroblasts. (A) Clustering of metabolic scores in fibroblasts from different sources. (B) Specific metabolic pathways in fibroblast cell clusters with different metabolic scores. (C) Correlation analysis between the top 10 genes and the top 3 pathways in fibroblast cell clusters (clusters) with different metabolic score clustering. (D) Differential expression of genes in key cellular pathways across various fibroblast metabolic subgroups. (E) Analysis of the relationship between fibroblast cell clusters with different metabolic scores and existing types of fibroblast cells. (F) Differences between fibroblast cell clusters with different metabolic scores and the main ACTA2+ and COL1A1+ fibroblast cells. (G) Transcription factor analysis (TF) in fibroblast cell clusters with different metabolic scores. (H) Tissue propensity analysis of fibroblast cell clusters with different metabolic classifications. (I) Cell communication analysis between fibroblast cell clusters with different metabolic scores and other cells in PVTT samples. (J) and (K) Activation levels of different metabolic fibroblast cell types in the same PVTT sample, scored using AUCcell; (J) Spatial visualization of activation levels in the sample; (K) Differences in activation levels among the four cell types.

In this study, we analysed 2199 CAFs derived from HCC, PVTT, and normal tissues (Supplemental Table S4 and Supplementary Figure S2A) and illustrated them in the UMAP plot (Figure 2A). Based on their metabolic pathway scores, we divided CAFs into four meta‐clusters (Fib‐C0, C1, C2, and C3). The top three metabolic pathways with the highest enrichment in each cell type are shown in Figure 2B. Notably, metabolism of polyamines was prominently active in both Fib‐C0 and C3 meta‐clusters. By correlating the dominant metabolic pathways with the key genes in each meta‐cluster, we constructed a network of metabolic pathways and top DEGs (Supplementary Figure S2B) to elucidate the potential influence of metabolic processes on transition of the TME of HCC (Figure 2C). Compared to Fib‐C0, C1, and C2 meta‐cluster, C3 meta‐cluster exhibited higher expression of cancer‐related pathway genes, including MMPs, TGF‐β, and collagen pathway genes (Figure 2D). Furthermore, using the established CAFs signatures, we observed that Fib‐C3 meta‐cluster had elevated proliferation scores (pan.pCAF) and collagen scores (pan.dCAF), as shown in Figure 2E. Additionally, Fib‐C3 meta‐cluster was characterized by a higher presence of COL1A1+ CAFs **(**Figure 2F, *p* <0.001**)**. TF analysis revealed several distinct TFs in the Fib‐C3 meta‐cluster, notably IRF2 and ELK4 (Figure 2G). Tissue preference analysis indicated that Fib‐C3 meta‐cluster had higher OR values in PVTT, whereas Fib‐C2 meta‐cluster was more prevalent in HCC tissues and Fib‐C1 meta‐cluster in normal tissues (Figure 2H and Supplemental Table S5). Moreover, Fib‐C3 meta‐cluster demonstrated increased cell communication links within PVTT tissue (Figure 2I, Supplementary Figure S2C, D). A PVTT spatial sample was used to detect the distribution of fibroblast cells (FAP+ cells), which confirmed that the top 50 gene signatures of Fib‐C3 meta‐cluster were higher than those of the other meta‐clusters (Figure 2J, K).

### 3.3 PVTT tissue‐specific myeloid metabolism promotes the M2 polarization

**FIGURE 3:**
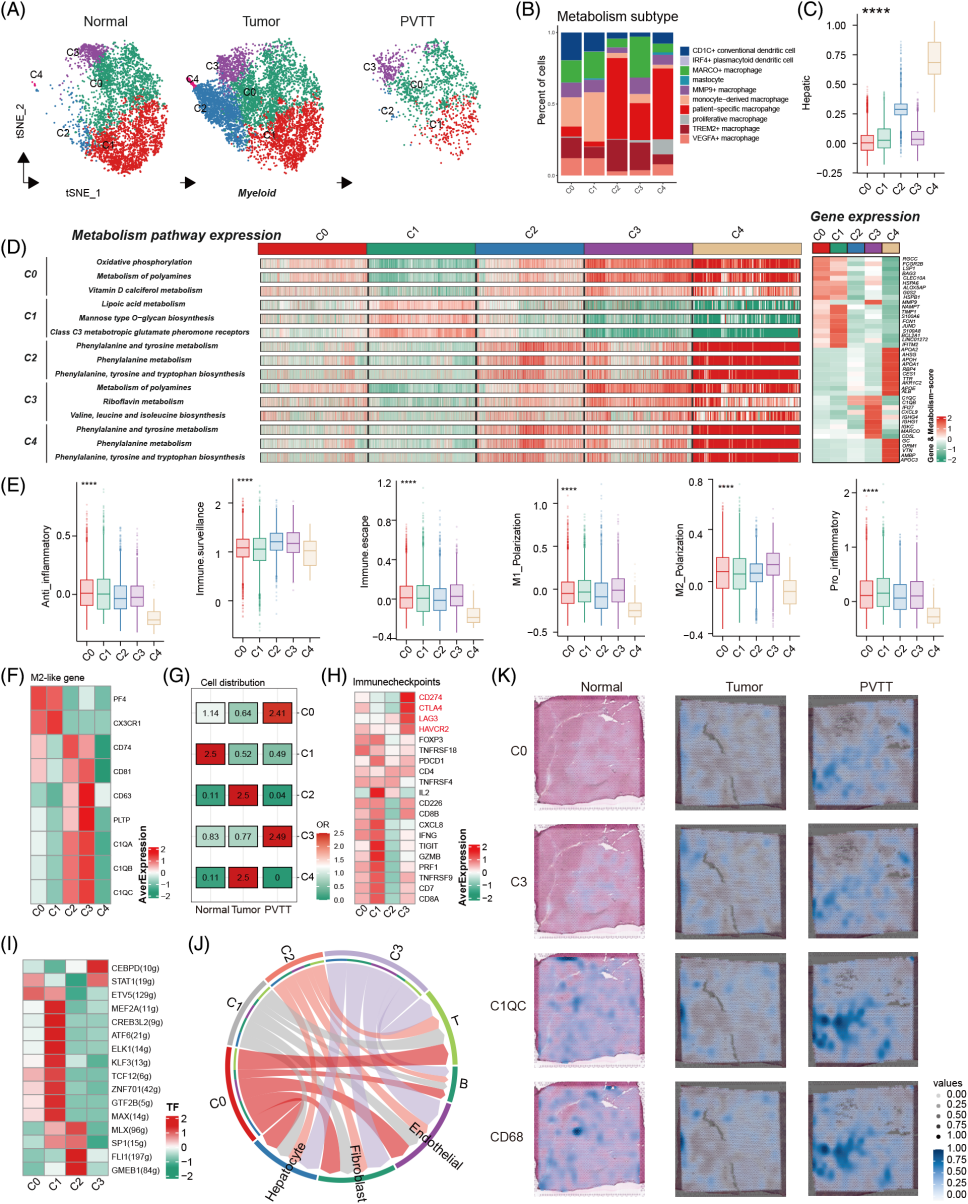
Metabolic heterogeneity analysis of Myeloid cells. (A) Classification of metabolic scores in myeloid cells from various sources. (B) Correlation analysis between the five metabolically classified myeloid cell types and common macrophages. (C) Detection of hepatic activity in myeloid cells using five different metabolic classifications. (D) Top three activated metabolic pathways and the top 10 genes in myeloid cells across 5 metabolic classifications. (E) Differences in inflammation, polarization scoring, and other functional scores in myeloid cells across the five metabolic classifications. (F) Expression of M2‐like associated genes in Myeloid cells across five metabolic classifications (Heatmap). (G) Tissue propensity analysis of myeloid cells according to the five metabolic classifications. (H) Differences in immune checkpoint expression in the four metabolically classified myeloid cell types in PVTT samples (heatmap). (I) Differences in transcription factor activation in four metabolically classified myeloid cell types in PVTT samples. (J) Changes in cell communication intensity between four metabolically classified myeloid cell types and other cells in the PVTT samples. (K) Expression of M‐C0, M‐C3, C1QC, and CD68 in three paired spatial transcriptomic samples.

For myeloid cells, we obtained and analysed a total of 14,990 individual cells. Using the metabolic characteristics (Supplementary Table S6 **)**, we divided them into five groups based on the significantly different numbers of cells for the Myeloid‐C0, C1, C2, C3, and C4 meta‐clusters (Figure 3A). Compared with the original cell annotation features, we observed significant differences among these clusters. The Myeloid‐C2, C3, and C4 meta‐clusters had excessive patient‐specific macrophages, which related with patient's genetic background,^24^, ^31^ whereas Myeloid‐C0 and C1 meta‐clusters had monocyte‐derived macrophages (Figure 3B). Notably, Myeloid‐C4 meta‐cluster exhibited a high hepatic score (Figure 3C), suggesting a diverse macrophage origin. We further analysed the top specific metabolic pathways in different subgroups and found that Myeloid‐C2, C3, and C4 meta‐clusters also enriched similar pathways, whereas the DEGs were completely different (Figure 3D). Within the previous signatures for myeloid features, we found that the C3 meta‐cluster had a high M2 polarization score and immune escape ability (Figure 3E). To check the expression of M2‐like genes in each cluster, we found that Myeloid‐C2 and C3 meta‐cluster had more corresponding genes such as C1QA, C1QB, and C2QC (Figure 3F). For tissue preference, we found that Myeloid‐C0 and C3 meta‐cluster were higher in the PVTT tissue, Myeloid‐C2 and C4 meta‐cluster were higher in HCC tissue, and only Myeloid‐C1 meta‐cluster was higher in the normal tissue (Figure 3G and Supplemental Table S7). In the PVTT samples, CD274 was highly expressed in Myeloid‐C3 meta‐cluster compared with the other meta‐clusters (Figure 3H). In addition, we detected the activity of TF for each meta‐cluster and found that CEBPD was the only TF in the Myeloid‐C3 meta‐cluster (Figure 3I). The cell–cell communication of myeloid cell metabolic meta‐cluster with other main cell types from different sources is listed in Supplemental Figure S3. Similar to Fib‐C3 meta‐cluster, Myeloid‐C0 and C3 meta‐cluster in PVTT had more links with other main cell types (Figure 3J). A patient with three paired spatial samples in the normal, HCC, and PVTT was used to detect the distribution of myeloid metabolism cluster cells (CD68+ cells). We annotated the Myeloid‐C0, C3, and two marker genes, CD68 and C1QC, in the spatial cells and found that PVTT had high expression of these cell activated scores and genes **(**Figure 3K **)**.

### 3.4 Metabolic heterogeneity of T cells exhibit different immune activities

**FIGURE 4:**
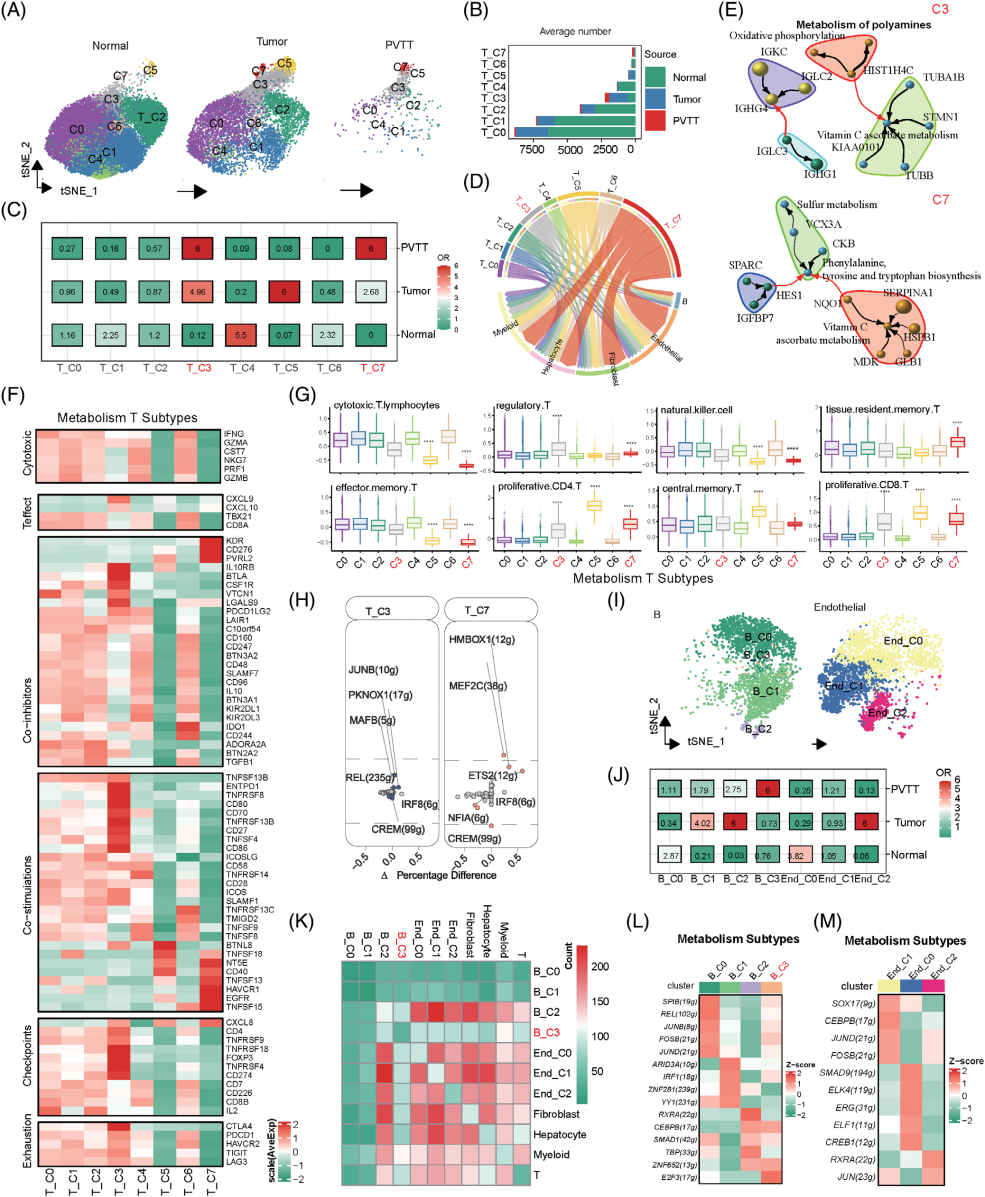
Analysis of metabolic heterogeneity in T, B, and endothelial cells. (A) Classification of metabolic scores of T‐cells from various sample sources. (B) Differences in cell numbers of the eight metabolically classified T cell types across different samples. (C) Tissue propensity analysis of the eight metabolic classifications of T cells. (D) Cell communication analysis between eight metabolically classified T‐cell types and other cells. (E) Correlation analysis of the top three metabolic pathways and top 10 genes in T‐C3 and T‐C7 cells. (F) Differential expression analysis of major immune genes in eight metabolically classified T‐cell types. (G) Differential expression of major cell‐type scores in eight metabolic classifications of T cells (symbols indicate significant differences between groups). (H) Key activated transcription factors in T‐C3 and T‐C7 cells. (I) Classification of metabolic scores in B and endothelial cells from various sample sources. (J) Tissue source propensity analysis of metabolic classifications in B and endothelial cells. (K) Cell communication analysis of B and endothelial cells in different metabolic states with other cells. (L) Differences in transcription factor activation among the four metabolic states of the B cells. (M) Transcription factor differences in the three metabolic states of endothelial cells.

Similar to the CAFs and myeloid cells, metabolic meta‐cluster method was performed on T cells, which generated eight metabolic meta‐clusters (Figure 4A and Supplementary Table S8). The results revealed substantial variations in the number of cells in different meta‐cluster across the different tissue types (Figure 4B and Supplementary Table S9). Organizational bias analysis indicated that T‐C3 meta‐cluster labelled with metabolism of polyamines, and T‐C7 meta‐cluster labelled with sulphur metabolism demonstrated a pronounced bias towards PVTT (Figure 4C). Notably, the T‐C7 meta‐cluster showed enhanced cellular communication in the PVTT samples, markedly surpassing the other meta‐clusters (Figure 4D). Furthermore, metabolic pathway and gene network analyses highlighted distinct metabolic features of T‐C3 and T‐C7 meta‐cluster (Figure 4E). The networks of meta‐clusters is detailed in Supplementary Figure S4A. Then, we performed differential expression analyses of immune genes (Supplementary Table S10) between T‐C3 and T‐C7 meta‐cluster and found that T‐C7 meta‐cluster lacked most of the co‐inhibitors, checkpoints, and effective and exhausted T‐related genes, which are present in T‐C3 meta‐cluster (Figure 4F). Metabolic clustering also correlated significantly with T cell annotation classes, exemplified by T‐C7 meta‐cluster marked downregulation in cytotoxicity, NK cell characteristics, and effector T cells, and upregulation in regulatory T cells, tissue‐resident memory T cells, and proliferative CD4 and CD8+ T cells **(**Figure 4G **)**. Additionally, TF analysis also showed differences in the C3 and C7 meta‐clusters (Figure 4H). Finally, cellphone analysis showed that ligand‐receptors of T cell meta‐clusters with other cells indicated marked disparities in PVTT samples (Supplementary Figure S4B, C).

### 3.5 Metabolic phenotypes of B cell and endothelial cell show metabolism source heterogeneity

We conducted metabolic clustering for both B and endothelial cells using identical resolution parameters, as shown in Figure 4I and Supplementary Table S11. Regarding their origin, we observed that B‐C3 meta‐cluster exhibited a predilection for PVTT, whereas cells expressing high levels in the tumour comprised of B‐C1, B‐C2, and End‐C2 meta‐cluster (Figure 4J and Supplementary Table S12). Additionally, analysis of cellular communication indicated marked disparities among B and endothelial meta‐cluster with others (Figure 4K). Furthermore, as illustrated in Figure 4L, M, the TF analysis of post‐clustering revealed substantial differences between B cells and epithelial cells.

### 3.6 Prognostic implications of TME metabolic subtypes

**FIGURE 5:**
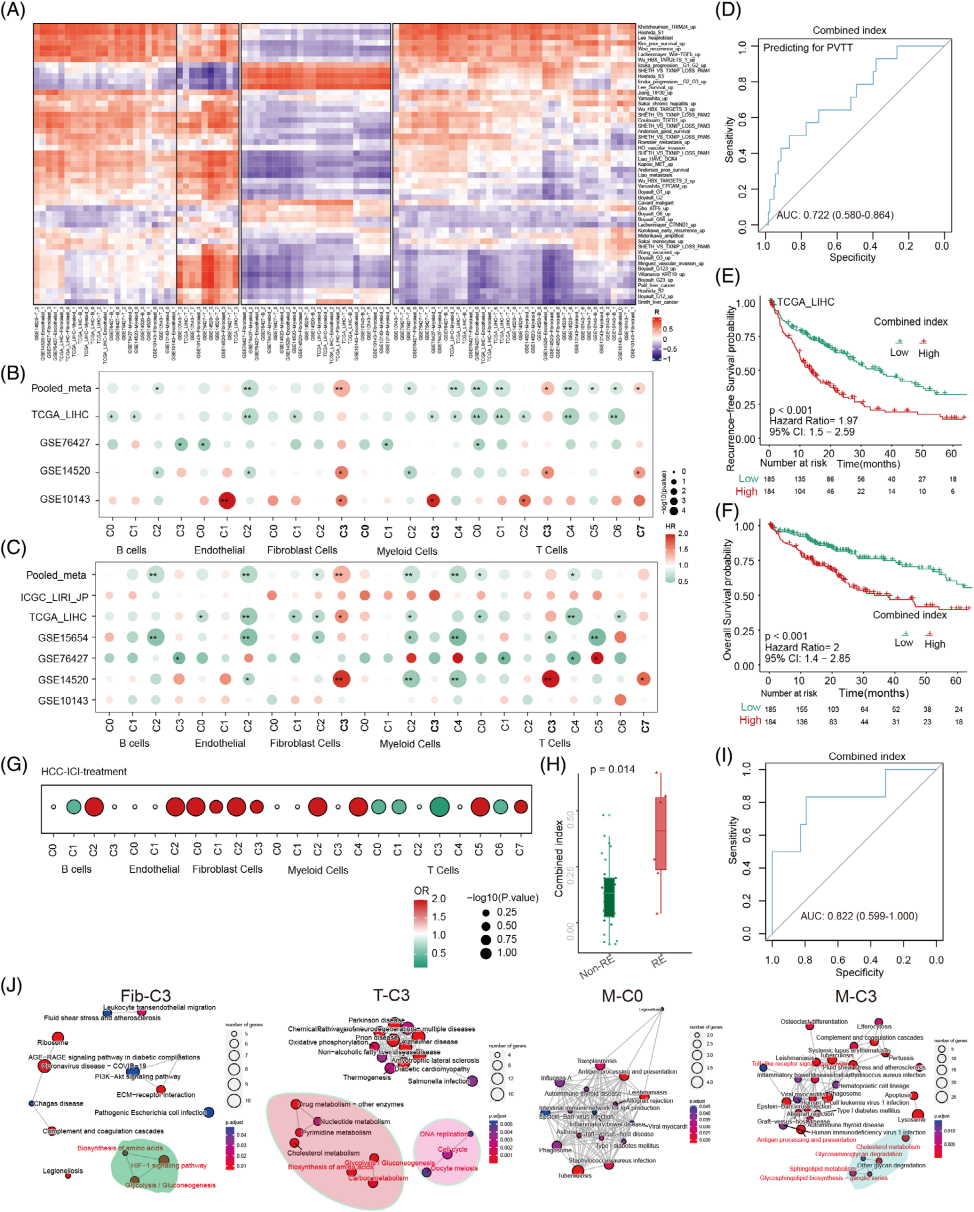
Clinical prognosis and therapeutic prediction value analysis of different metabolic classifications in non‐TME cells. (A) Correlation analysis between all metabolic classifications and common malignant tumour pathways in six public datasets. (B) Analysis of all metabolic classifications with overall survival (OS) in liver cancer across the four public datasets. (C) Analysis of all metabolic classifications with recurrence‐free survival (RFS) in liver cancer across the six public datasets. (D) ROC analysis of the combined scoring of cells with high metabolic expression in PVTT for predicting PVTT occurrence (from TCGA‐LIHC). (E) Prediction of liver cancer RFS using the combined scoring of cells with high metabolic expression in PVTT. (F) Prediction of liver cancer OS using the combined scoring of cells with high metabolic expression in PVTT. (G) Prediction of immune response in liver cancer patients who underwent immunotherapy based on all metabolic classification scores. (H) Expression differences in the combined scoring of cells with high metabolic expression in PVTT between liver cancer patients with and without an immune response. (I) ROC analysis for predicting the immune response in liver cancer using combined scoring of cells with high metabolic expression in PVTT. (J) Functional enrichment scoring of key PVTT cells with high metabolic expression levels.

To check the prognosis value of the TME metabolic subtype in HCC with PVTT, we firstly sought 43 HCC prognosis signatures from MSigDB (Supplementary Table S13) and subsequently examined the associations between these HCC prognosis scores and metabolic subtype scores in six bulk RNA sequencing data of HCC (Figure 5A). All marked genes of these meta‐clusters of non‐malignant cells in HCC are listed in Supplementary Table S14. Remarkably, in the context of high expression in PVTT, T‐C3, T‐C7, Myeloid‐C0, and Fib‐C3 meta‐clusters showed significant alignment with these adverse HCC‐related signatures. Conversely, Myeloid‐C2, Myeloid‐C4, and most T‐cell meta‐clusters displayed minimal correlation with these signatures (Figure 5A). A meta‐analysis was performed to evaluate the prognostic implications of these meta‐clusters in HCC across multiple cohorts. Regarding recurrence‐free survival (RFS), Fib‐C3, T‐C3, and T‐C7 meta‐clusters were identified as contributors to HCC progression (Figure 5B and Supplementary Table S15). However, for overall survival (OS), only Fib‐C3 meta‐cluster demonstrated a notable negative impact (Figure 5C and Supplementary Table S16). Intriguingly, by focusing on cell subtypes with high expression in PVTT including Fib‐C3, Myeloid‐C0, Myeloid‐C3, T‐C3, and T‐C7 meta‐clusters, we discerned a distinctive influence on PVTT occurrence using logistic regression analysis (AUC, 0.722; 95%CI = 0.580–0.864, Figure 5D). This combined index also provided effective predictions for the OS and RFS of HCC patients in the TCGA‐LIHC cohort **(**Figure 5E, F *p* <0.001**)**.

### 3.7 Evaluating of metabolic subtypes for immunotherapeutic response in cancers

To investigate the role of metabolically active cells and their response to immunotherapy in HCC patients with PVTT treated with immune checkpoint inhibitors, we employed logistic regression model to examine the relationship between meta‐clusters (Fib‐C3, Myeloid‐C0, Myeloid‐C3, T‐C3, and T‐C7) and RECIST response outcomes. This revealed that an increased score of cell metabolic phenotype genes correlates with a change in immunotherapy response in HCC patients (Figure 5G). Notably, the composite combined index of meta‐clusters in PVTT demonstrated significantly differential expression between the response (RE) and non‐response (non‐RE) group (Figure 5H). The ROC analysis indicated that the area under the curve (AUC) of the combined index to predict the immunotherapy response was 0.822 (95%CI 0.599–1.000) (Figure 5I). Furthermore, we extended our analysis to evaluate the relevance of these metabolically active PVTT cells in other cancers treated with immunotherapy, such as metastatic melanoma, urothelial carcinoma, and bladder cancer, as depicted in Supplementary Figure S5A. ROC analysis corroborated the similar predictive efficacy of the combined index, with a range of 0.65–0.71, as detailed in Supplementary Figure S5B. Beyond metabolic pathway characterization, our investigation encompassed GO function analysis and KEGG pathway analysis of the predominant genes in these single cell meta‐clusters. We identified several common tumour‐associated pathways in Fib‐C3, T‐C3, and Myeloid‐C3 meta‐clusters, including glycolysis/gluconeogenesis and amino acid biosynthesis, as indicated in Figure 5J and Supplementary Figure S5C, D.

### 3.8 Co‐expression of metabolism of polyamines in TME cells of PVTT promotes the occurrence of PVTT

**FIGURE 6:**
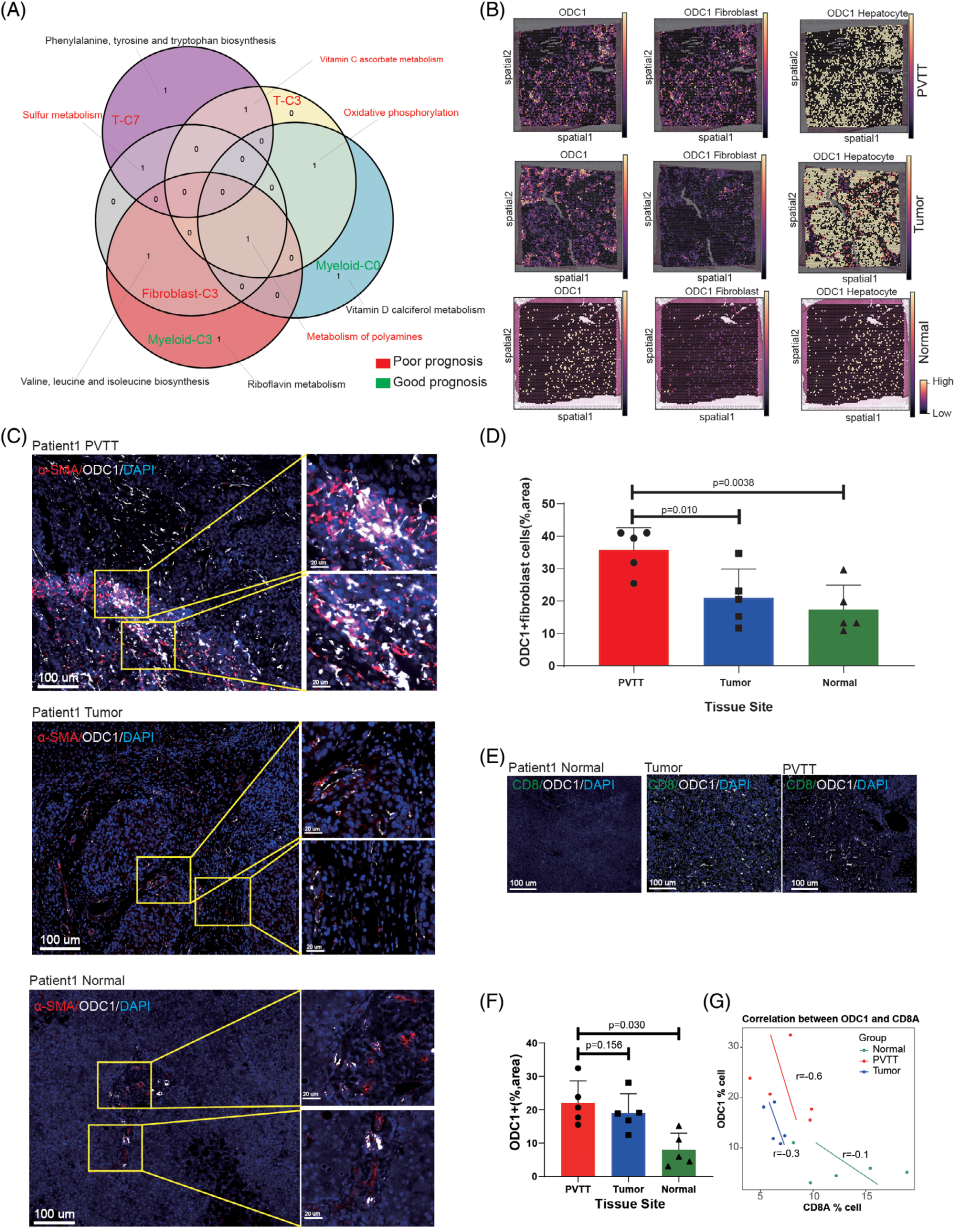
Discovery and validation of co‐expressed metabolic pathways in PVTT non‐TME cells. (A) Cross‐analysis of metabolic pathways in different high‐expression cells in the PVTT (Venn diagram). (B) Cell2location show the spatial co‐expression of metabolism of the key polyamine enzyme ODC1 with Fibroblast and hepatocyte cells in spatial samples. (C) mIHC validation of α‐SMA/ODC1 co‐expression in normal, tumour, and PVTT samples. (D) Differential analysis of α‐SMA+/ODC1+ cells in normal, tumour, and PVTT samples. (E) Co‐expression analysis of ODC1 and CD8T cells in normal, tumour, and PVTT samples. (F) Differential expression analysis of ODC1+ cells in normal, tumour, and PVTT samples. (G) Correlation analysis of ODC1 and CD8A in normal, tumour, and PVTT samples.

To identify common highly expressed metabolic pathways in TME cells of PVTT samples, we employed co‐expression analysis for these meta‐clusters. The top three metabolic pathways were selected among the five cell types (Fib‐C3, Myeloid‐C0, Myeloid‐C3, T‐C3, and T‐C7 meta‐clusters). Using Venn diagrams, we observed similarities in the metabolic pathway activation across these meta‐clusters. For instance, metabolism of polyamines was highly expressed in Fib‐C3, Myeloid‐C0, and Myeloid‐C3 meta‐clusters, whereas oxidative phosphorylation was predominant in Myeloid‐C0 and T‐C3 meta‐clusters. Additionally, sulphur metabolism was notably expressed in T‐C7 and Fib‐C3 meta‐clusters (Figure 6A). Focusing on the key gene Ornithine Decarboxylase 1 (ODC1) in the metabolism of polyamines pathway in Fib‐C3 meta‐clusters, we conducted a spatial transcriptomics sequencing for further validation. We examined the spatial distribution of ODC1 in spatial transcriptome sequencing of PVTT and its co‐expression with CAFs and hepatocyte cells. In tissue samples, the positivity rate of ODC1 in the CAFs of PVTT was significantly higher than that in HCC and normal samples (Figure 6B). In contrast, the positivity rate of ODC1 in PVTT hepatocytes was similar to that in HCC samples and higher than that in normal samples (Figure 6B). Subsequently, we performed fluorescent mIHC on 15 samples from five paired PVTT‐HCC‐normal samples. These results further confirm our findings (Figure 6C). Paired *t*‐tests revealed statistically significant differences in the expression of ODC1+ fibroblasts in normal, HCC, and PVTT samples (Figure 6D, 35% vs. 21% vs. 17%, *p* <0.05). Moreover, we observed that ODC1 expression was significantly higher in PVTT and HCC tissues than in normal tissues (Figure 6E, F), and identified a negative correlation between ODC1 and CD8A (Figure 6G).

## 4 DISCUSSION

The utility of various sequencing technologies for understanding the underlying mechanisms of tumorigenesis has been revealed and validated.^32^ Accumulating evidence has shown that the metabolic abnormality could contribute to the initiation and progression of malignant tumours including HCC.^33^, ^34^ Understanding the extent and detailed metabolic heterogeneity of HCC and PVTT is crucial because it could predict outcomes in HCC patients with PVTT and guide therapeutic strategies. Our study is the first to present comprehensive metabolic landscape analysis of non‐malignant cells in HCC and PVTT through multi‐omics analysis of large‐sample, multicenter sequencing data. Our findings deepen the knowledge of non‐malignant cells within the TME and extend the understanding of metabolic landscape of HCC and PVTT.

Poor prognosis and low response to treatment with PVTT remain common challenges.^35^, ^36^, ^37^ Few studies have demonstrated the metabolic heterogeneity in HCC and PVTT, which may pose pivotal challenges for developing effective treatment strategies. For this status, we explored six major cells including T, B, myeloid, endothelial cells, CAFs, and hepatocytes in the TME to reveal the metabolic heterogeneity among HCC, PVTT, and normal liver tissues. We found significant differences in the metabolic pathway scores among these six primary cell types, such as riboflavin, D‐d‐arginine, and d‐ornithine metabolism and so forth, which have been verified to be widely involved in tumour metabolism and affect the malignant progression of tumours.^38^, ^39^, ^40^ Next, we depicted the different metabolic landscapes within non‐malignant cells in HCC, PVTT, and normal liver tissues. Compared to HCC and normal tissues, PVTT had distinct metabolic landscape, particularly in CAFs and T cells. This showed a significant conversion of metabolic heterogeneity among the non‐malignant PVTT cells.

Previous studies have demonstrated that CAFs can modulate cancer cell growth, progression, and evasion from cancer therapies and act as active participants in the complex metabolism of tumours.^15^, ^22^ Our study first categorized CAFs into four meta‐clusters (Fib‐C0, C1, C2, and C3), and revealed the top three metabolic pathways with the highest expression in each cluster. Compared to other clusters, we found that cluster Fib‐C3 exhibited a higher correlation with tumour biology, which was also verified using spatial transcriptomics sequencing data. These findings demonstrate that CAFs have dynamic plasticity during HCC and PVTT progression, and cluster Fib‐C3 indicates poor prognosis and therapeutic efficacy, which is in accordance with previous research. It is a noteworthy strategy to make use of CAFs plasticity for the conversion of different clusters to affect the component in the TME, and finally improve the therapeutic efficiency.

Myeloid cells are the most abundant components in the TME, where they exert a variety of functions including tumour metabolism and immunosuppression.^41^, ^42^ The myeloid cells are composed of many different cell types, including monocytes, macrophages, dendritic cells and granulocytes.^43^ Among these cell types, TAMs are considered pivotal components that contribute to tumour proliferation and progression.^43^, ^44^, ^45^ The results showed that Myeloid‐C2, C3, and meta‐clusters had excessive patient‐specific macrophages, and Myeloid‐C0 and C3 meta‐clusters were involved in the metabolism of polyamines. In addition, we found that the Myeloid‐C3 meta‐cluster had a high anti‐inflammatory (M2) polarization score. Previous studies have demonstrated that TAMs can alter polarization into pro‐inflammatory (M1) and M2 phenotypes, depending on the TME.^46^ M2 polarization of TAMs promotes the proliferation and metastasis of various tumours^47^, ^48^ and can exert immunosuppressive effects to reduce the efficacy of immunotherapy.^49^ Recent studies using scRNA sequencing analysis have comprehensively delineated TAMs heterogeneity in HCC.^50^, ^51^ Our study also found that Myeloid‐C0 and C3 meta‐cluster were enriched in HCC patients with PVTT. Therefore, For Myeloid‐C3 meta‐cluster PVTT, the response to immunotherapy is relatively poor. Our findings had a certain ability to guide the implementation of classification and treatment strategies for PVTT patients.

A growing body of research on metabolic abnormalities of T cells has demonstrated obvious heterogeneity of immune cell distribution in HCC.^52^ For instance, it has been reported that increased glycolytic metabolism is related to resistance to immunotherapy.^53^ Several recent studies have demonstrated that targeting specific aspects of tumour‐intrinsic metabolism, such as the hexosamine biosynthesis pathway or glutamine metabolism, can foster an immune response and sensitize tumours to checkpoint blockade.^17^, ^54^ In our study, we found that T‐C3 meta‐cluster labelled with metabolism of polyamines and T‐C7 meta‐cluster labelled with sulphur metabolism demonstrated a pronounced bias towards PVTT. Notably, the T‐C7 meta‐cluster showed enhanced cellular communication. We also found that T‐C7 meta‐cluster lacked most co‐inhibitors, checkpoints, and effective and exhausted T cell‐related genes. This could also explain the relatively poor effectiveness of immunotherapy in HCC patients with PVTT.

Remarkably, Fib‐C3, Myeloid‐M0, Myeloid‐M3, T‐C3, and T‐C7 meta‐clusters were significantly enriched in PVTT and were significantly aligned with adverse liver cancer signatures. Highly enriched Fib‐C3, T‐C3, and T‐C7 meta‐clusters indicated shorter RFS in patients with HCC. However, only Fibroblast‐C3 meta‐cluster demonstrated a notable negative impact on the OS of patients with HCC. Therefore, to facilitate the clinical usage of metabolic clusters, we integrated five meta‐clusters, Fib‐C3, Myeloid‐M0, Myeloid‐M3, T‐C3, and T‐C7, to develop a combined index. This combined index discerned a distinctive influence on PVTT occurrence and provided effective predictions for OS and RFS in HCC patients. Cheng et al. study constructed a CT‐based radiomics nomogram to predict OS of HCC patients with PVTT, the C‐index for the radiomics model was 0.759 in the training cohort and 0.730 in the validation cohort. In the present study, the C‐index of our risk score was higher than Cheng et al.'s model.^55^

Given the recent establishment of cancer immunotherapy, including the use of blocking antibodies against immune checkpoint pathways, several studies have begun to establish a relationship between tumour‐intrinsic metabolism and successful immunotherapy.^56^ In the present study, we revealed that the combined index of metabolic cells in PVTT was significantly different between responder and non‐responder groups. Patients with a high combined‐index exhibited a sensitivity response to immunotherapy. This implies a combined index that might guide immunotherapy for HCC patients with PVTT. However, given that the survival analysis, patients often have poor prognoses without immunotherapy, and patients can benefit significantly with immunotherapy.

As previously mentioned, metabolism of polyamines is considered a key metabolic pathway involved in the conversion of metabolic heterogeneity in HCC and PVTT. Thus, we detected and validated a key gene, ODC1, in the metabolism of polyamines pathway by spatial distribution analysis and mIHC technology. ODC1, a key rate‐limiting enzyme, is a poor prognostic indicator for HCC and other tumours.^57^ In line with the results of a previous study, ODC1 expression was significantly higher in HCC patients with PVTT.^58^ We also found a negative correlation between ODC1 and CD8A expression. At present, few researchers explored the role of ODC1 in the tumour immunotherapy response. Thus, by conducting further investigations of ODC1, it is hopeful target to enhance the effectiveness of immunotherapy.

Despite its comparative advantages, our study has certain limitations. First, the present study deeply explored the impact of metabolic heterogeneity on the development and therapeutic strategy of PVTT, but there is a lack of research on the impact of metabolites on PVTT and the interpretation of spatial level. In addition, some of the data used in this study comes from public databases; therefore, selection bias is inevitable. Therefore, future research efforts should be devoted to confirming the applicability and efficacy of the cluster approach and the combined index using more extensive patient cohorts.

In conclusion, this study for the first time conducted a comprehensive analysis of metabolic heterogeneity in non‐malignant cells at the multi‐omics level. Our study reveals both consistency and heterogeneity in the metabolism of non‐malignant cells in HCC patients with PVTT. The risk stratification based on CAFs and Myeloid cells conduces to predict prognosis and guide treatment. We also explore the key role of polyamine and sulphur metabolism in immune cell function. These findings offer new directions for understanding PVTT development and immunotherapy response.

## AUTHOR CONTRIBUTIONS

All authors read and approved the final version of the manuscript. *Study concept and design*: YZ‐G, RL, MG‐H, XP‐Z. *Drafting of the manuscript*: XP‐Z, WB‐Z, ZQ‐L, ZT‐Y. *Acquisition of data, analysis, and interpretation of data*: YZ‐G, XP‐Z, WB‐Z, ZQ‐L, ZT‐Y. *Critical revision of the manuscript*: YZ‐G, RL. *Statistical analysis*: SB‐Y, ZY‐L, FF‐W, PJ‐L. *Study supervision*: YZ‐G, RL, MG‐H. All authors read and approved the final manuscript.

## FUNDING INFORMATION

This study was supported by the National Natural Science Foundation of China (No. 32201232); Sponsored by Beijing Nova Program (20230484372); Young Elite Scientists Sponsorship Program by CAST (2023QNRC001); Capital Health Development Research Special Project (2024–4‐5026); Young Elite Scientists Sponsorship Program by BAST (No. BYESS2024001); National Key Research and Development Program of China (2022YFC2407402); the Fundamental Research Funds for the Central Universities of HIT.

## CONFLICT OF INTEREST STATEMENT

The authors declare that they have no known competing financial interests or personal relationships that could have influenced the work reported in this study.

## CONSENT TO PARTICIPATE

Written informed consent was obtained from all the patients.

## CONSENT FOR PUBLICATION

Consent for publication was obtained from all the authors.

## Supporting information


**Figure S1:** (A) UMAP visualization depicting the metabolism scores of the patients. (B) UMAP visualization illustrating metabolic clusters in normal, tumour, and PVTT samples. (C) Metabolic phenotypes and their quantities in various sample types.
**Figure S2:** (A) Activation of metabolic pathways within different fibroblast cell clusters. (B) Representative differentially expressed genes in different fibroblast metabolic clusters. (C) Variations in cell communication ligand‐receptor co‐expression between different fibroblast metabolic clusters and other cells (heatmap). (D) Cell communication ligand‐receptor co‐expression between different fibroblast metabolic clusters and other cells (dot plot).
**Figure S3:** (A)–(C) Changes in cell communication co‐expression quantity between myeloid cells from normal, tumour, and PVTT samples and other cells (heatmap). (D) Cell communication ligand‐receptor co‐expression between different myeloid cell metabolic clusters and other cells (dot plot).
**Figure S4:** (A) Correlation between the top three metabolic pathways and the top 10 genes in the T cell metabolic clusters. (B) Changes in cell communication ligand‐receptor quantities between T cell metabolic clusters and other cells (heatmap). (C) Cell communication ligand‐receptor co‐expression between T cell metabolic clusters and other cells (dot plot).
**Figure S5:** (A) Relationship between major metabolic clusters and immune responses in non‐HCC patient cohorts. (B) ROC analysis predicting the immune response using the combined score of cells highly expressing metabolic clusters in PVTT. (C) Functional analysis of metabolic clusters in different non‐tumour cells (GO enrichment analysis). (D) Pathway enrichment analysis of metabolic clusters in non‐tumour cells.

**Table S1:** Variations in metabolic pathway scores across different cell types in liver cancer.
**Table S2:** Differences in metabolic pathway scores in non‐tumour cells from various sources.
**Table S3:** Variation of metabolic pathway scores in non‐tumour cells across different tissue origins.
**Table S4:** Differences in metabolic pathway scores between different fibroblast cell metabolic clusters.
**Table S5:** Tissue distribution preferences analysis of different fibroblast cell metabolic clusters.
**Table S6:** Variations in metabolic pathway scores between different myeloid cell metabolic clusters.
**Table S7:** Tissue distribution preferences analysis for different myeloid cell metabolic clusters.
**Table S8:** Differences in metabolic pathway scores among different T cell metabolic clusters.
**Table S9:** Tissue distribution preferences analysis for different T cell metabolic clusters.
**Table S10:** List of relevant immune gene names.
**Table S11:** Differences in metabolic pathway scores between different B and End cell metabolic clusters.
**Table S12:** Tissue distribution preferences analysis for different B and End cell metabolic clusters.
**Table S13:** Gene markers for non‐tumour cell metabolic clusters in liver cancer.
**Table S14:** List of names for liver cancer‐related malignant pathways (MsiDB names).
**Table S15:** Overall survival (OS) prognosis analysis for non‐tumour cell metabolic clusters in liver cancer.
**Table S16:** Recurrence‐free survival (RFS) prognosis analysis for non‐tumour cell metabolic clusters in liver cancer.

## Data Availability

The data that support the findings of this study are available from the corresponding author upon reasonable request.
