# Supplementary material for: The heterogeneity of cellular metabolism in the tumour microenvironment of hepatocellular carcinoma with portal vein tumour thrombus
Source: Cell Prolif. 2024 Aug 27;58(1):e13738. doi: 10.1111/cpr.13738 (PMC11693549; doi:10.1111/cpr.13738)
Supplement: Supplementary file 1 — Figure S1: (A) UMAP visualization depicting the metabolism scores of the patients. (B) UMAP visualization illustrating metabolic clusters in normal, tumour, and PVTT samples. (C) Metabolic phenotypes and their quantities in various sample types. Figure S2: (A) Activation of metabolic pathways within different fibroblast cell clusters. (B) Representative differentially expressed genes in different fibroblast metabolic clusters. (C) Variations in cell communication ligand‐receptor co‐expression between different fibroblast metabolic clusters and other cells (heatmap). (D) Cell communication ligand‐receptor co‐expression between different fibroblast metabolic clusters and other cells (dot plot). Figure S3: (A)–(C) Changes in cell communication co‐expression quantity between myeloid cells from normal, tumour, and PVTT samples and other cells (heatmap). (D) Cell communication ligand‐receptor co‐expression between different myeloid cell metabolic clusters and other cells (dot plot). Figure S4: (A) Correlation between the top three metabolic pathways and the top 10 genes in the T cell metabolic clusters. (B) Changes in cell communication ligand‐receptor quantities between T cell metabolic clusters and other cells (heatmap). (C) Cell communication ligand‐receptor co‐expression between T cell metabolic clusters and other cells (dot plot). Figure S5: (A) Relationship between major metabolic clusters and immune responses in non‐HCC patient cohorts. (B) ROC analysis predicting the immune response using the combined score of cells highly expressing metabolic clusters in PVTT. (C) Functional analysis of metabolic clusters in different non‐tumour cells (GO enrichment analysis). (D) Pathway enrichment analysis of metabolic clusters in non‐tumour cells. [file CPR-58-e13738-s001.docx]

Supplementary figures

**Title:**

**The heterogeneity of cellular metabolism in the tumor microenvironment of hepatocellular carcinoma with portal vein tumor thrombus**

**
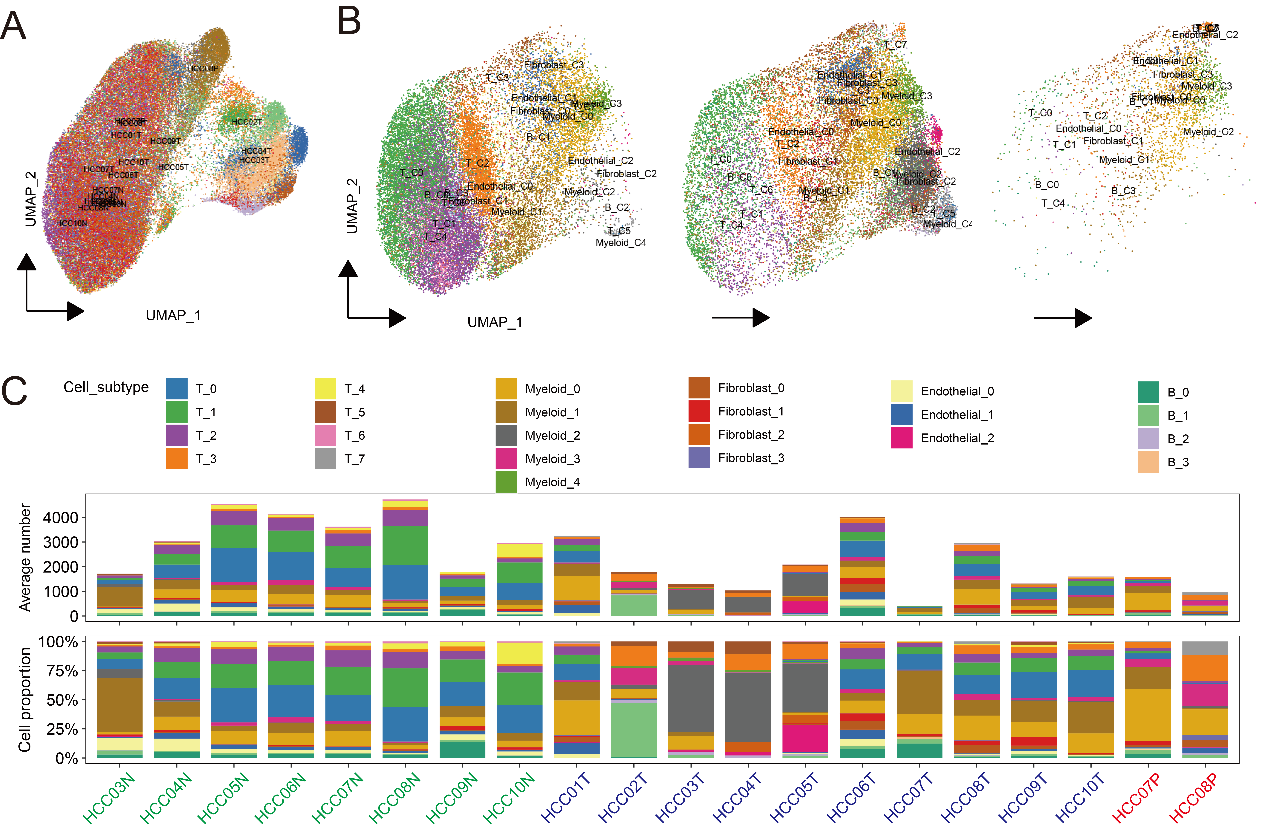
**

**Figure S1:** A) UMAP visualization depicting the metabolism score in patients. B) UMAP visualization illustrating metabolism clusters in normal, tumor, and PVTT samples. C) Metabolic phenotypes and their quantities in various sample types.


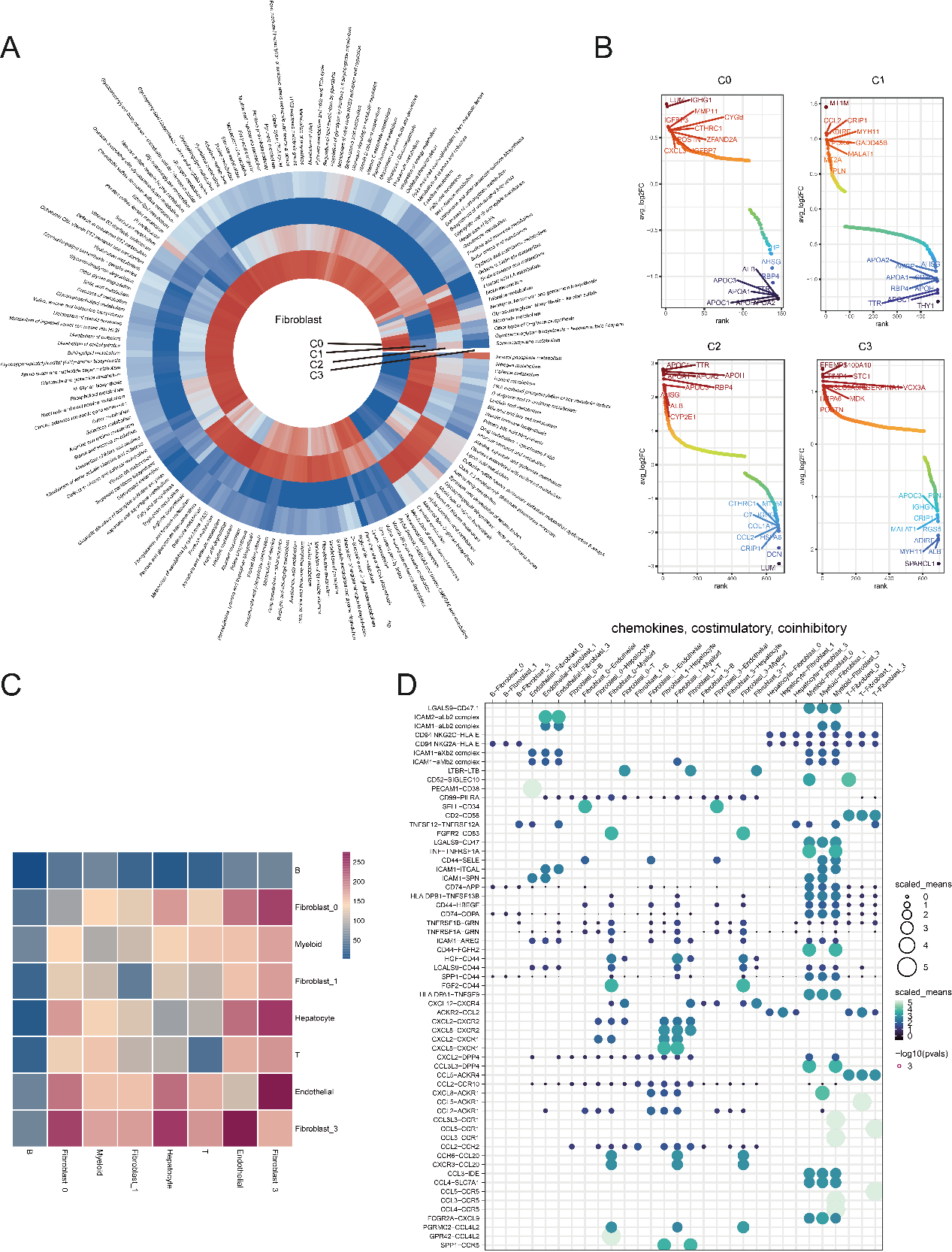


**Figure S2:** A) Activation of metabolic pathways within different fibroblast cell metabolic clusters. B) Representative differential genes in different fibroblast metabolic clusters. C) Variations in cell communication ligand-receptor co-expression between different fibroblast metabolic clusters and other cells (heatmap). D) Cell communication ligand-receptor co-expression between different fibroblast metabolic clusters and other cells (dot plot).


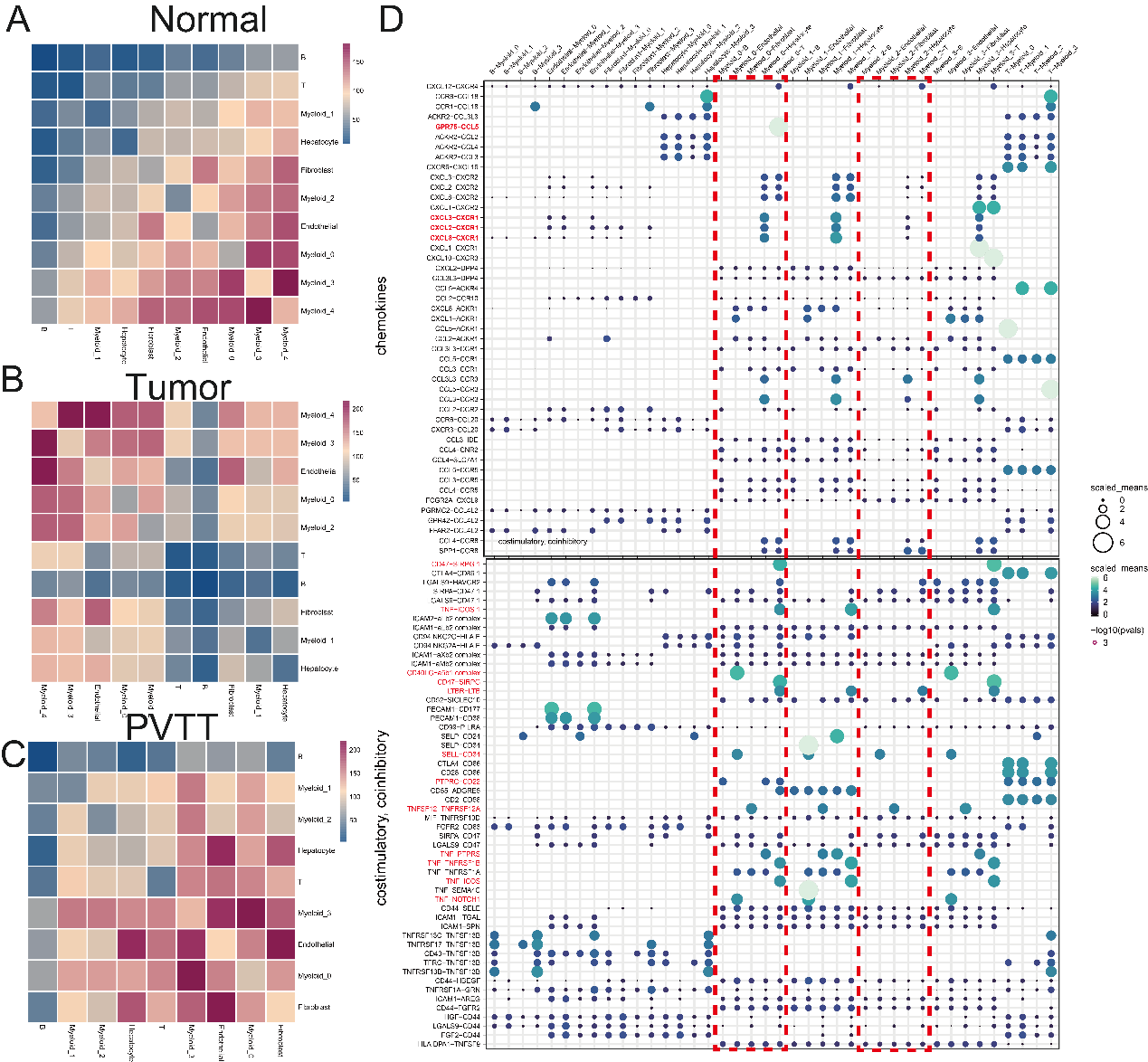


**Figure S3:** A), B), and C) Changes in cell communication co-expression quantity between myeloid cells from normal, tumor, and PVTT samples and other cells (heatmap). D) Cell communication ligand-receptor co-expression between different myeloid cell metabolic clusters and other cells (dot plot).


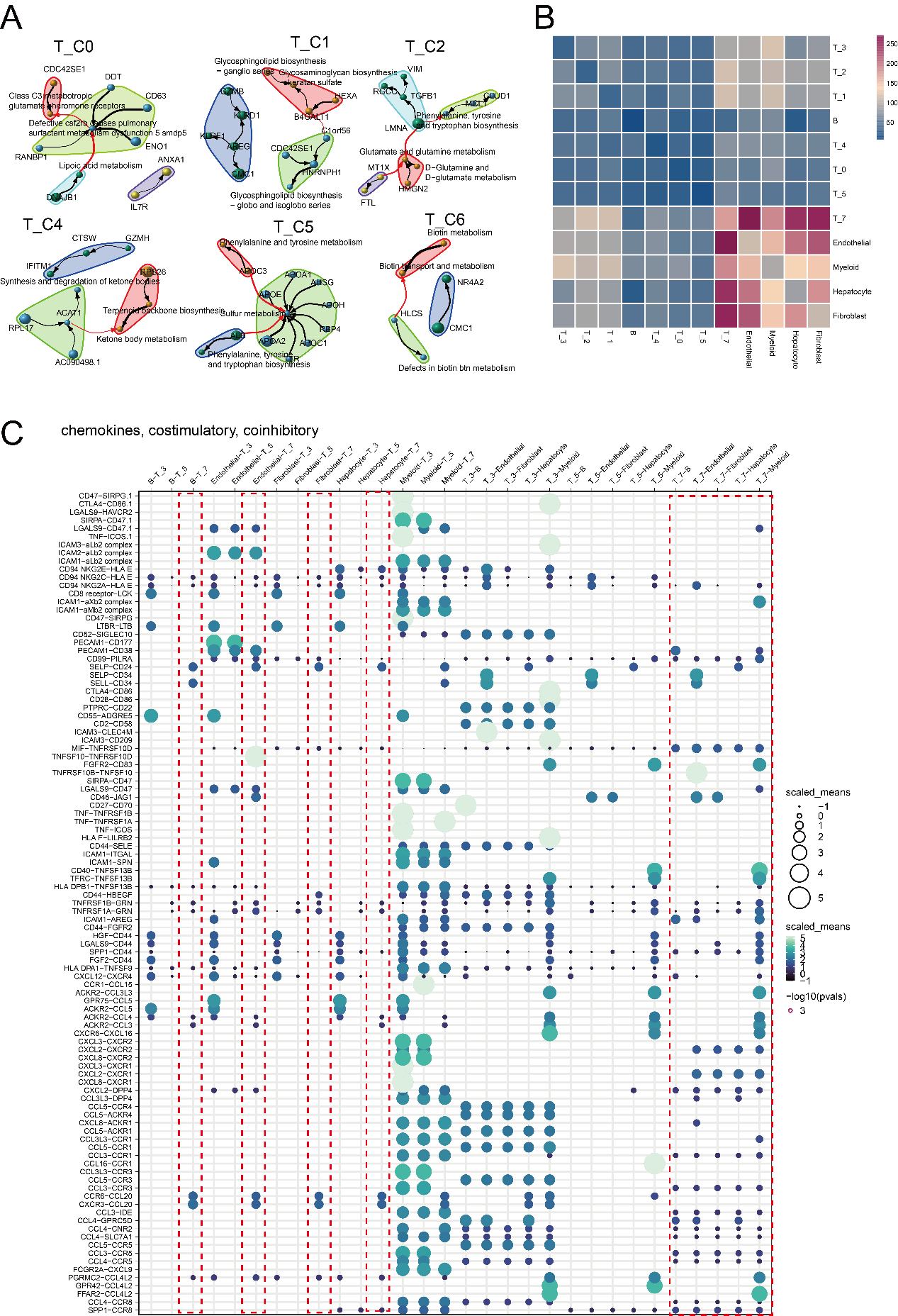


**Figure S4:** A) Correlation between the top 3 metabolic pathways and the top 10 genes in T cell metabolic clusters. B) Changes in cell communication ligand-receptor quantity between T cell metabolic clusters and other cells (heatmap). C) Cell communication ligand-receptor co-expression between T cell metabolic clusters and other cells (dot plot).

**
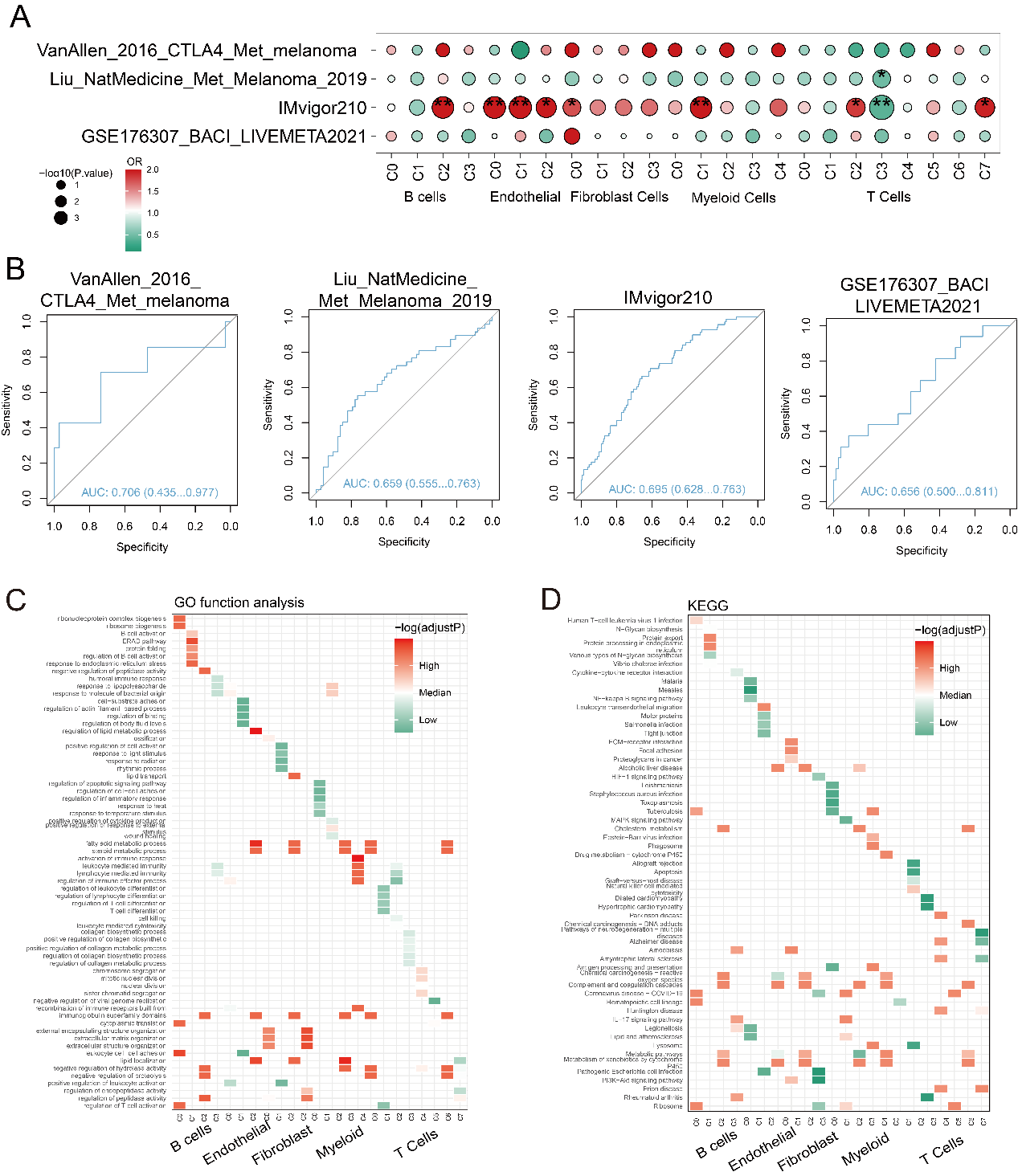
Figure S5:** A) The relationship between major metabolic clusters and immune response in non-hepatocellular carcinoma patient cohorts. B) ROC analysis predicting immune response using the combined score of cells highly expressing metabolic clusters in PVTT. C) Functional analysis of metabolic clusters in different non-tumor cells (GO enrichment analysis). D) Pathway enrichment analysis of metabolic clusters in different non-tumor cells.
